# Russian military bloggers: Corpus and dataset of collected posts, 2022–2023

**DOI:** 10.1016/j.dib.2025.112015

**Published:** 2025-09-04

**Authors:** Marco Albertini, Giampiero Giacomello, Aidar Zinnatullin

**Affiliations:** a Computational Social Science Center (CSSC), Department of Political and Social Sciences, University of Bologna, Italy; b Rheinland-Pfälzische Technische Universität Kaiserslautern-Landau, Germany

**Keywords:** Telegram, Russia, War in Ukraine, Topic modeling, Milbloggers

## Abstract

The ongoing war in Ukraine has generated unprecedented volumes of digital data across multiple domains, from weapons effectiveness and battlefield casualties to military communications and economic impacts. This digitalization of warfare has created new opportunities for computational social science research, paralleling the broader trend of increasing digital traces in human activities. Our study focuses on the communications and subsequent reactions within Russian military bloggers' (``milbloggers'') Telegram channels, specifically examining their war-related content in the original Russian langiage.

These communications have emerged as a crucial component of the Russian information sphere, potentially influencing war policy and political decision-making. Telegram's perceived security features, including robust encryption and privacy protocols, have made it a preferred platform for such discourse. The Russian leadership has strategically utilized milbloggers' posts and their audience reactions as both a barometer of public sentiment and a tool for shaping public opinion regarding the conflict's developments.

While quantifying the effectiveness of these influence operations remains challenging due to data limitations, our analysis concentrates on the observable ``input'' - the milbloggers' posts and their associated reactions. This dataset provides valuable insights into the ongoing conflict and serves as a foundation for modeling public opinion dynamics and sentiment evolution in digital warfare contexts.

Specifications Table

**Table:** 

Subject	Social Sciences
Specific subject area	*Political Science, Computational Social Science, text-as-data, NLP*
Type of data	Tables in CSV and RData formats.
Data collection	*The dataset was collected after the queries made to the official API of Telegram to get the information about the publicly available Telegram channel of the most popular Russian pro-war military bloggers.* *We queried the official Telegram API via Telethon to collect complete post histories for 21 public “milblogger” channels. We first relied on the investigative journalists’ curated list (*[1]*; The* [2]*), which was constructed from TGStat statistics, and then verified subscriber counts and inter-channel citations directly on TGStat* [3]*. We retained channels that (i) were public, (ii) consistently posted on the war, and (iii) were active in our study window. For each channel, we used the method messages.GetHistoryRequest to page through all available posts and stored the raw message objects (JSON). No date, keyword, or language filters were applied to the collection. After collection, we generated post-level language predictions with three detectors in R (textcat, cld2, cld3) and released these variables in the dataset to document the language of each post and enable optional filtering. The raw corpus spans November 6, 2016–October 17, 2023 (448,742 posts).*
Data source location	*The dataset collected from*https://telegram.org/
Data accessibility	***Please note:****All raw data referred to in this article must be made publicly available in a data repository prior to publication. Please indicate here where your data are hosted (the URL must be working at the time of submission and editors and reviewers must have anonymous access to the repository):* Repository name:Open Science Framework Data identification number: DOI 10.17605/OSF.IO/CTBZ9 Direct URL to data: https://osf.io/ctbz9/
Related research article	*none*

## 1 Value of the Data

• This dataset can be helpful for researchers in political science to analyze the content of pro-war military bloggers on the Telegram platform in Russia, providing insights into narratives and framing used to shape public opinion on the war.
• The dataset can be valuable for social scientists researching political communication, propaganda, and the sociology of conflict, offering a basis for understanding the spread of battlefield information and ideological messaging.
• The dataset can be used to train and evaluate machine learning models to automate the identification of key topics, sentiments, and spread patterns of the pro-war content, aiding experts in the study of information warfare and its impacts.
• These data were collected from Telegram channels, then analyzed and filtered, and can serve as a benchmark dataset for other researchers in political science, communication studies, and computational social sciences, particularly in the context of military and propaganda studies.

## 2 Background

The 2022 Russian invasion of Ukraine hardened political repression within Russia. Authorities intensified crackdowns on dissent, targeting independent media and foreign social platforms first. Amid this clampdown, a new group of military bloggers—“voenkory”—emerged to document battlefield events, using Telegram [4], a major hub for political debate in Russia. Although united in supporting the invasion, these bloggers differ in their level of radicalism and criticism of the Defense Ministry’s tactical choices. Their growing prominence influences war coverage, resonates in mainstream media, and indirectly affects decisions on the battlefield. By shaping public perceptions, they also bolster pro-war narratives, posing significant challenges for political stability. Understanding this “digital arena” is therefore critical to grasp how media discussions and government policies shape each other. Moreover, analyzing this dataset illuminates the broader ramifications of pro-war narratives for both democracy and international peace, underscoring the importance of scrutinizing these emerging forms of influence.

## 3 Data Description

We provide the data for the 21 most popular Telegram channels according to the number of subscribers and the citation by other channels (source: TGStat^1^). We mostly rely on the list curated by independent Russian journalists who investigate the activity of military bloggers (for instance, the Russian version^2^ of the investigation about the authors of one of the most influential channels, Rybar [1], and its English version^3^ (The [2]). Overall, the raw data corpus contains 448,742 posts and covers the period from the 6th of November 2016, because some channels started functioning long before the full-scale invasion of Ukraine. At the same time, we provide a brief exploratory text analysis of the content relevant only to the period from February 21, 2022,^4^ until October 17, 2023. We end the window there because our interest is regime dynamics during the war, and the Prigozhin mutiny (June 2023) and his death (late August 2023) mark pivotal moments after which the Kremlin consolidated tighter control over the Telegram ecosystem. Extending the end date several weeks beyond his death lets us observe immediate post-event adjustments in how military bloggers, including the Prigozhin-affiliated channel GREY ZONE [5] in our corpus, reported on the war, capturing potential changes in tone, framing, and topical emphasis that followed these developments. The version of the dataset used for exploratory text analysis contains 293,954 Telegram posts. Table 1 presents the list of Telegram channels within the scope of the dataset and the descriptive study.

**Table 1:** The list of military bloggers.

№	Title	Subscribers⁎
1	Рыбарь (Rybar)	1104,819
2	Мир сегодня с “Юрий Подоляка” (World Today with Yuri Podolyaka)	2751,841
3	WarGonzo	1319,385
4	Операция Z: Военкоры Русской Весны (Operation Z: Military bloggers of the Russian Spring)	1169,137
5	Сладков + (Sladkov +)	1009,061
6	Поддубный |Z|О|V| edition (Poddubny ZOV edition)	862,398
7	Colonelcassad	844,411
8	Стрелков Игорь Иванович (Strelkov Igor Ivanovich)	792,194
9	Архангел Спецназа Z (Archangel of special forces unit Z)	739,007
10	Kotsnews	670,235
11	Повёрнутые на Z войне (Turned on the Z war)	648,298
12	Александр Ходаковский (Aleksandr Khodakovsky)	632,383
13	Старше Эдды (Older than Edda)	625,936
14	Владлен Татарский (Vladlen Tatarsky)	539,336
15	Военный осведомитель (Military informer)	530,823
16	МИГ России (MIG of Russia)	461,479
17	GREY ZONE	436,109
18	Vоенкор Котенок Z (Military Blogger Kotenok Z)	418,528
19	Неофициальный Безсонов ``Z'' (Unofficial Bezsonov Z)	388,376
20	ANNA NEWS	334,236
21	Захар Прилепин (Zakhar Prilepin)	287,001

⁎ Number of subscribers by February 2023.

Rather than snowball sampling, we rely on a curated sampling frame tailored to prominent pro-war Russian “milblogger” channels. This choice aligns the population with our substantive focus (pro-Kremlin actors) and enhances transparency and replicability. Snowball procedures on Telegram are useful for discovery (see [6,7]) but are vulnerable to non-random message deletion on Telegram, which biases content and network inferences and worsens with time (with text in groups and forwards/videos in channels especially affected) [8]. Given these known challenges and our substantive interest, we prioritize a curated, high-salience panel and provide the full list so that future work can augment it with snowball expansion when a broader ecosystem map is the goal.

To contextualize our contribution, several public Telegram resources on the Russia–Ukraine war exist but differ in purpose and scope. Relative to the Center for Urban History’s Telegram Archive of the War [9], our corpus is narrower and analysis-ready. This archive^5^ is an emergency preservation project that contains a very broad mix of channels and group chats, primarily Ukrainian sources, but also Russian propaganda, at a large scale (1048 channels; around 28 TB), with changing thematic priorities and a curated access model. Our dataset, by contrast, targets a specific slice of the Telegram ecosystem related to Russia’s domestic political conversation: 21 public, high-salience pro-war “milblogger” channels that map onto different actors and media holdings inside the regime. This includes channels affiliated with Prigozhin/Wagner networks (e.g., GREY ZONE), tabloid-linked media (Komsomolskaya Pravda’s Kotsnews), and state broadcaster ecosystems (VGTRK-related bloggers such as Сладков+ [Sladkov+] and Поддубный |Z|О|V| [Poddubny ZOV]).

Compared to the “Shaping the Narratives of the Russia-Ukraine War for Western Audiences” dataset [10], which curates 55 verified English-language Telegram channels about the war, our release is complementary rather than overlapping. Their focus is English-language outreach and Western-facing discourse, whereas our list is explicitly oriented to Russian-language, domestically salient pro-Kremlin channels that influence intra-elite narratives and public opinion inside Russia.

In relation to TeleScope [11], which offers the largest longitudinal Telegram dataset to date (metadata for >500k channels and detailed message metadata for around 71k public channels with 120 M messages) but is not war-specific, our contribution is depth over breadth. TeleScope is ideal for macro-level, cross-domain analyses; our corpus is a curated, domain-specific panel tailored to pro-war Russian political communication, letting researchers study narrative competition among distinct regime-adjacent actors (e.g., military correspondents, state media personalities, and Wagner-aligned outlets).

The dataset obtained after the queries made to the official API of Telegram contains variables:

• message: The content of the message shared in the Telegram channel.
• date: The date and time when the message was posted in the channel.
• views: The number of views the message has received within the channel.
• number_replies: The number of replies to the message within the channel's thread.
• number_forwards: The number of times the message has been forwarded by channel followers.
• is_forward: A Boolean variable indicating whether the message is a forwarded message from another channel (True) or an original one (False).
• forward_msg_date: The date and time when the original forwarded message was posted.
• forward_msg_date_string: A string representation of the forward message's date and time.
• is_reply: A Boolean variable indicating whether the message is a reply to another message within the channel.
• contains_media: A Boolean variable indicating whether the message contains any media (True or False).
• media_type: The type of media contained in the message, such as image, video, document, etc.
• has_url: A Boolean variable indicating whether the message contains a URL link.
• url: The URL included in the message.
• domain: The domain of the URL included in the message (e.g., ``example.com'').
• url_title: The title of the webpage linked through the URL.
• url_description: A short description of the webpage linked through the URL.
• document_type: The type of document (if applicable) shared in the message, such as PDF, DOCX, etc.
• video_duration_secs: The duration of the video shared in the message (if applicable), measured in seconds.
• poll_question: The question presented in a poll (if the message includes a poll).
• poll_number_results: The number of responses or results collected in the poll.
• textcat: Post-level language predicted by R’s textcat (character n-gram classifier). Value is the best-matching language–encoding profile string (e.g., “russian-koi8_r”, “russian-windows1251”, “bulgarian-iso8859_5”) rather than a pure ISO code; you can normalize to the base language by removing the encoding suffix.
• cld2: Post-level language predicted by cld2 (Google CLD2). Value is the most-likely ISO-639 code (e.g., “ru”, “en”, “uk”); may return special script-only tags of the form “xx-*” (e.g., “xx-Qaai”, “xx-Runr”) when only a script is detected.
• cld3: Post-level language predicted by cld3 (Google CLD3, neural). Value is a BCP-47/ISO-639 language tag, optionally with a script variant (e.g., “ru”, “uk”, “ru-Latn”, “zh-Latn”).

## 4 Experimental Design, Materials and Methods

### 4.1 Explorative text analysis

Data collection and filtering. We interfaced with Telegram using Telethon (Python), authenticated via the official API. For each channel handle listed in Table 1, we resolved the entity and iteratively called messages.GetHistoryRequest^6^ (page size to return is 100) until the server returned an empty page, saving the resulting message dictionaries to disk with ISO-8601 timestamps. After retrieval, we computed post-level language predictions using three detectors in R (i.e., textcat [12], cld2 [13], cld3 [14]) and wrote these predictions into the shared dataset as variables named textcat, cld2, and cld3. So, we did not apply any language filter at the collection stage, and the language-related columns allow transparent auditing and optional exclusion rules by others if desired.

We use structural topic modeling (STM) [15] to derive the most prevalent topics in the posts of pro-war military bloggers. STM allows for the inclusion of control variables to estimate corresponding topical content and topical prevalence. Topical content pertains to the likelihood of specific words appearing within a given topic, while topic prevalence signifies the ratio of topics in individual documents within a corpus. For this STM model, we used the Telegram channel as a covariate. Such variables as views, number_replies, and number_forwards are highly informative for analyzing user engagement. Researchers using our dataset can incorporate these engagement measures as document-level prevalence covariates to assess how topic proportions vary with engagement, thereby measuring how users interact with different topics in the messages, for example, identifying which topics systematically attract more views, replies, or forwards after accounting for channel and posting time.

Topic modeling required conducting text pre-processing steps such as tokenization, conversion to lowercase, deletion of punctuation, stopwords,^7^ special characters, emojis, and numbers. Then, all the words were lemmatized, i.e., converted to their base form since, for the Russian language, lemmatization is more efficient for the performance of topic models than stemming (removal of the word ending) (May, Cotterell, & Durme, 2016). To do so, we exploited the MyStem program created by Yandex.^8^ We removed words that appear in fewer than one document (lower.thresh parameter of the prepDocuments command in the STM package), but we did not specify the upper bound because the list of stopwords is quite extensive (*n* = 568). Then, we did not sample the corpus but analyzed all the available Telegram posts due to available computational capacities. We also removed forwarded messages from other channels.

One of the challenges commonly encountered in topic modeling is determining the number of topics for the model (k-value). The aim of choosing a k-value is to yield topics which are both semantically meaningful and distinguishable from one another. We follow the iterative approach implemented by Bossetta, Segesten, & Bonacci [16] and based on suggestions made by other researchers [15,17] to detect an appropriate number of topics. We exploited the searchK function from the STM package [15] to generate a set of models with diagnostics parameters. Specifically, we orient on the balance between semantic coherence and exclusivity. Semantic coherence evaluates the frequency with which the most defining words of a topic co-occur. Exclusivity examines whether words in a topic are unique to that topic or are distributed across multiple topics. These metrics are instrumental in arriving at a topic model with interpretable topics that are clearly delineated from one another.

For choosing a k-value, we first set the interval of 10 within the range of 10 and 100. Subsequently, we narrowed the spectrum, focusing on the range of 5 and 30 (both values included) with the interval of 5. Finally, we checked the interval between 11 and 20 (both included). The most favorable balance between semantic coherence and exclusivity is provided when k-value is equal to 15.

## 5 Results of Topic Modeling

**Fig. 1:**
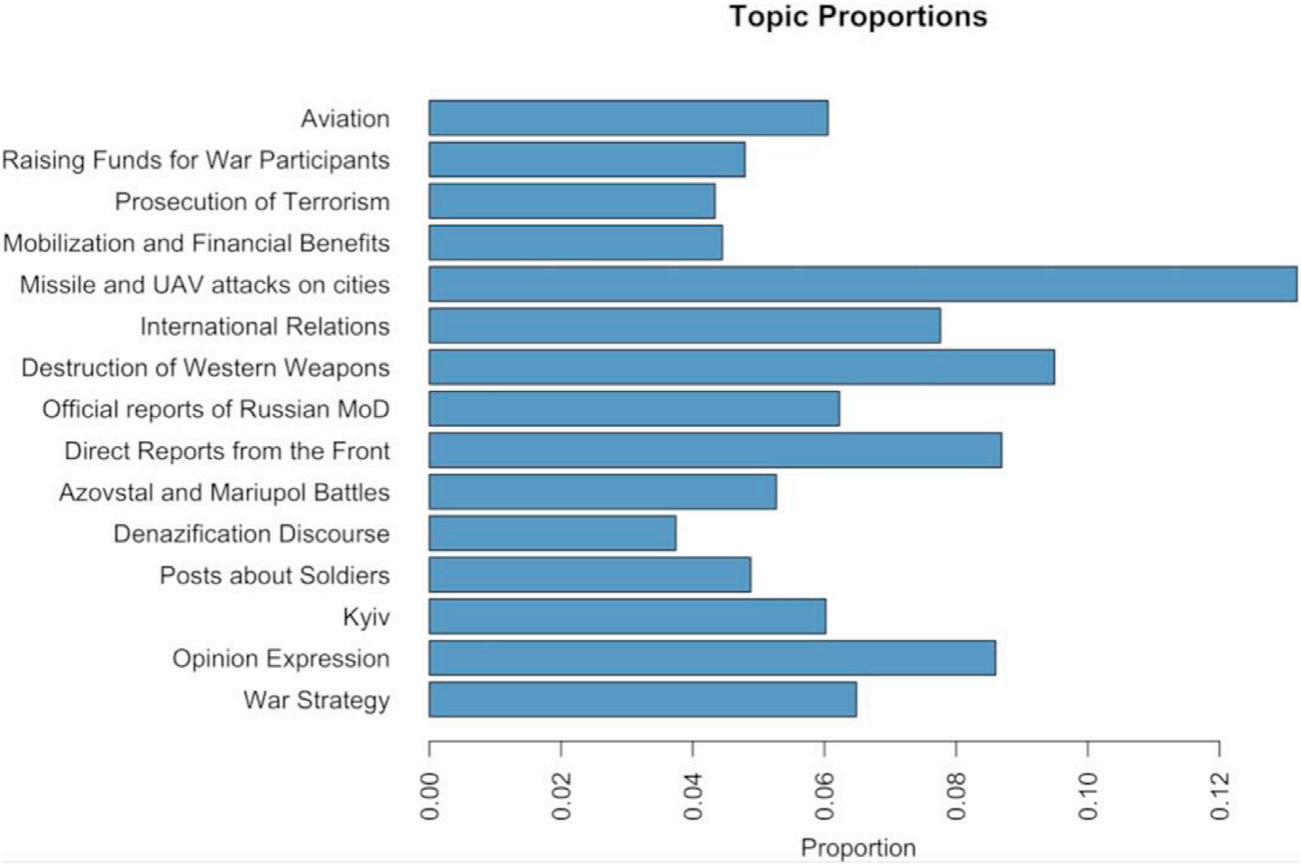
Topics in Telegram posts.

Fig. 1 provides an overview of the most prevalent topics in the posts, while Table 2 presents the words with the highest probability score, translated into English for all 15 topics. The summary of the topics was labeled after a careful reading of the representative Telegram posts.

**Table 2:** Summary of STM of the Telegram posts circulated by pro-war military channels, 15 topics, focus on the highest probability words.

Topic (Label)	Highest Probability Words	Translation to English	Share
1 (War Strategy)	проблема, ситуация, возможность, действие, западный, количество, данный	problem, situation, opportunity, action, western, quantity, given	6 %
2 (Opinion Expression)	начинать, подписываться, воевать, кстати, хохол, точно, интересный	start, sign up, fight, by the way, Russian, exactly, interesting	9 %
3 (Kyiv)	киев, сообщать, военный, заявлять, информация, видео, источник	Kyiv, report, military, declare, information, video, source	6 %
4 (Posts about soldiers (awarding Russians and messages with a list of Ukrainian prisoners of war))	боец, донбасс, бригада, боевой, командир, батальон, подразделение	fighter, Donbass, brigade, combat, commander, battalion, unit	5 %
5 (Denazification Discourse)	победа, история, флаг, родина, великий, брат, язык	victory, history, flag, homeland, great, brother, language	4 %
6 (Azovstal and Mariupol Battles)	мариуполь, боевик, наемник, мирный, житель, израиль, убивать	Mariupol, militant, mercenary, civilian, resident, Israel, kill	5 %
7 (Direct Reports from the Front)	направление, противник, бой, войско, район, позиция, наступление	direction, enemy, battle, army, area, position, offensive	9 %
8 (Official reports of the Ministry of Defense of the Russian Federation)	уничтожать, район, пункт, военный, направление, техника, боеприпасы	destroy, area, point, military, direction, equipment, ammunition	6 %
9 (Destruction of Western Weapons)	видео, танк, противник, кадр, позиция, уничтожать, враг	video, tank, enemy, frame, position, destroy, enemy	9 %
10 (International Relations)	сша, заявлять, нато, президент, поддерживать, конфликт, американский	USA, declare, NATO, president, support, conflict, American	8 %
11 (Missile and UAV attacks on cities)	область, удар, район, объект, взрыв, наносить, обстрел	area, blow, area, object, explosion, inflict, shelling	13 %
12 (Mobilization and Financial Benefits)	военный, путин, владимир, президент, подготовка, мобилизация, служба	military, Putin, Vladimir, president, training, mobilization, service	4 %
13 (State Prosecution of Terrorism, Espionage, Mutiny)	власть, гражданин, территория, организация, сотрудник, террористический, задерживать	power, citizen, territory, organization, employee, terrorist, detain	4 %
14 (Raising Funds for War Participants)	канал, помощь, проект, средство, помогать, подписчик, карта	channel, help, project, means, help, subscriber, map	5 %
15 (Aviation)	ракета, военный, самолет, мост, пво, крым, море	rocket, military, plane, bridge, air defense, Crimea, sea	6 %

As can be seen from Fig. 1, the most popular topic in Telegram posts is direct reports from the frontline. Here is an example of such posts:

URGENT NM DPR clears Volnovakha

This is reported by sources of the @wargonzo project on the front line.

Advanced Donbass units have already entered Volnovakha directly and have begun clearing the city. @wargonzo^9^

Also, most often, pro-war Russian Telegram channels publish materials with official reports prepared by the Russian Ministry of Defence. Here is an example of such posts:

Briefing by the official representative of the Russian Defense Ministry Igor Konashenkov as of 11.00 June 11, 2022 on the progress of the special operation in Ukraine

The Armed Forces of the Russian Federation continue a special military operation in Ukraine.

In the ANDREEVKA area of the Kharkov region, high-precision air-launched missiles destroyed the deployment point of foreign mercenaries.

In addition, over the past 24 h, 9 areas of concentration of manpower and military equipment of the Ukrainian Armed Forces, five firing positions of units of multiple launch rocket systems in the areas of the settlements MALORYAZANTSEVO, VOLCHEYAROVKA, PODGORNOE, MALYA ILYINOVKA and LISICHANSK of the Lugansk People's Republic were hit, and an anti-aircraft missile system was destroyed ``Buk-M1″ in the MINKOVKA area of the Donetsk People's Republic…^10^

In addition, messages promoting the official discourse of denazification of Ukraine’s political elite and army are prevalent in the corpus of Telegram posts. Here is an example of a post framing the conflict in this way:

Russian Ministry of Defense: Kyiv ordered militants in Mariupol to shoot those who want to lay down their arms

Surrounded and completely blocked in Mariupol on the territory of the Azovstal metallurgical plant, the Ukrainian group was asked to voluntarily lay down their arms and surrender in order to save their lives.

“However, the Kiev nationalist regime, according to radio interception data, prohibited negotiations on surrender, ordering the Azov Nazis to shoot on the spot anyone who wanted to lay down their arms among Ukrainian military personnel and foreign mercenaries,” the Ministry of Defense said.^11^

In the list of topics from the Telegram channels under study (Fig. 1), there is also a topic that we call “Culture war.” These are posts about Russian celebrities (musicians, writers, actors) who spoke out against the war in Ukraine, as well as messages promoting authors who decided to actively support the Russian invasion with their work:

The place where Vanya Urgant sat is still empty. Ivan. Well, Ivan is on sabbatical for now. May he have a good time. Why is the place empty? People are used to it.

They would have taken Stas Starovoitov to host an Evening with Starovoitov. Instead of Anton Dolin, Mikhail Trofimenkov, the best film critic in Russia, would talk about cinema there. And then Anton left somewhere. Instead of Galya Yuzefovich, who won’t go on TV now, let Alexey Kolobrodov talk about books, and let Karaulov and Pegov and Revyakina and Dolgareva read poetry. Instead of Grebenshchikov, let Dmitry Revyakin bring his new records, and instead of Kinchev, the same Kinchev will come. And instead of Husky - Husky. Neither of them left.

It will be a good program.

Not?

Well, that’s right, otherwise suddenly everyone who left would return, and someone was sitting in their chair, eating from a bowl, touching a spoon.

“Let’s keep everything as it was under grandma.”

Meanwhile, you can do normal, smart, patriotic, in a good rhythm, fashionable, witty TV program. Can. There would be a desire.^12^

## Limitations

Telegram is a privately held company (no publicly traded shares), founded in 2013 by the by Nikolai and Pavel Durov(who is also the CEO), two Russian brothers who had also founded VKontakte (VK), popular Russian social networking platform. The preference for Telegram's channel and message communications over other platforms such WhatsApp or Instagram (both owned by Meta) rests on two key differences, namely ownership of the platforms and (claomed) greater security and privacy of Telegram, albeit the architecture and features of the named apps as well as others are different, and none is perfectly secure. It is crucial to understand that “more secure” is relative and depends on the specific threat model, in the case of Telegram vs. for example Instagram/WhatsApp is likely related to the ownership of the platforms, with the latter belonging to Mr. Mark Zuckenberg (US citizenship).

The perceived superior “security” of Telegram and, in the case of Russian milbloggers and users, its ownership have made Telegram the preferred platform to comment, post, and share information about the most diverse issues related to the war in Ukraine and its consequences. Overall, Telegram’s security is based on 1) client-side encryption for “secret chats” that use end-to-end encryption, namely, only the sender and receiver can read the messages, whereas Telegram's servers do not have access to the content. WhatsApp also offers end-to-end encryption, but its implementation and overall security posture have been subject to more scrutiny and debate; 2) the presence of open-source clients, so that, while the core protocol is not fully open-source, Telegram does allow for the development of third-party clients. This means independent security researchers can scrutinize the client-side code, potentially uncovering vulnerabilities more quickly than with a closed-source system like WhatsApp. Nonetheless, it is important to remember that using unofficial clients does not automatically exclude abuses and scams; 3) the option for self-destructing messages, as Telegram allows for setting a timer on messages, after which they automatically disappear from both the sender and receiver's devices (now WhatsApp allows those too). This adds an extra layer of privacy for sensitive information; 4) more granular control over metadata, that is, while metadata (as whom a person is communicating with and when) is still collected by both platforms, Telegram arguably offers users more granular (i.e., more detailed) controls over what data to share and privacy settings. Hence, while specific content may not be accessible, metadata can still reveal patterns of communication, which is equally important for law enforcement and government agencies.

It is worth noting, finally, that the company is registered in the British Virgin Islands and has an operational center in Dubai (UAE) that has different data privacy laws than many other jurisdictions. This affects how easily data can be requested by government authorities. With its better reputation in some “security” areas and the Russian ownership have undoubtedly made Telegram the preferred “unofficial” government-friendly communication and information channel in Russia.

## Ethics Statement

The authors have complied with the ethical requirements for publication in Data in Brief and confirm that this work does not involve human subjects or animal experimentation. The dataset comprises content from public Telegram channels only, and the released files include no personal identifiers, user-level metadata, or channel-level identifiers. All user-linked fields, such as Telegram user IDs, phone numbers, usernames, profile links, and message-entity fields that resolve to individual users, as well as channel usernames/handles and channel IDs were removed prior to release; only post content with derived, non-identifying features is provided. Data are distributed in accordance with Telegram’s data-redistribution policies and remain compliant with GDPR Recital 26.

## Credit Author Statement

**Aidar Zinnatullin:** Conceptualization, Methodology, Software, Writing – original draft, Investigation; **Giampiero Giacomello:** Conceptualization, Validation, Writing – review & editing; **Marco Albertini:** Conceptualization, Validation, Writing – review & editing.

## Data Availability

OSFRussian Military Bloggers: Corpus and Dataset of Collected Posts (Original data).

## Footnotes

1 https://tgstat.ru/en/ratings/channels.

2 https://thebell.io/kto-vedet-voennyy-telegram-kanal-rybar-rassledovanie-the-bell.

3 https://en.thebell.io/unmasking-russia-s-influential-pro-war-rybar-telegram-channel/.

4 On that day, Russian President Putin announced that the Russian government would recognize the Ukrainian separatist regions of the Donetsk People's Republic and the Luhansk People's Republic as independent.

5 https://telegram.lvivcenter.org.

6 https://core.telegram.org/method/messages.getHistory.

7 The source of Russian stopwords is the following: https://raw.githubusercontent.com/stopwords-iso/stopwords-ru/master/stopwords-ru.txt.

8 MyStem https://yandex.ru/dev/mystem/, accessed 21 March 2024.

9 Translation made by Google Translate. The link to the original post in Russian: https://t.me/wargonzo/6070, accessed 26 April 2024.

10 Translation made by Google Translate. The translated post has been shortened. The link to the original post in Russian:https://t.me/rybar/33805, accessed 26 April 2024.

11 Translation made by Google Translate. The link to the original post in Russian: https://t.me/sashakots/31337, accessed 26 April 2024.

12 Translation made by Google Translate. The link to the original post in Russian: https://t.me/zakharprilepin/17259, accessed 26 April 2024.
